# Increased titers of neutralizing antibodies after immunization with both envelope proteins of the porcine endogenous retroviruses (PERVs)

**DOI:** 10.1186/1743-422X-9-260

**Published:** 2012-11-05

**Authors:** Joachim Denner, Debora Mihica, Danny Kaulitz, Christa-Maria Schmidt

**Affiliations:** 1 Center of HIV and Retrovirology, Robert Koch Institute, Nordufer 20, 13353, Berlin, Germany

**Keywords:** Vaccine, Neutralizing antibodies, HIV-1, Porcine Endogenous Retroviruses (PERV)

## Abstract

Despite enormous difficulties to induce antibodies neutralizing HIV-1, especially broadly neutralizing antibodies directed against the conserved membrane proximal external region (MPER) of the transmembrane envelope protein, such antibodies can be easily induced in the case of gammaretroviruses, among them the porcine endogenous retroviruses (PERVs). In addition to neutralizing antibodies directed against the transmembrane envelope protein p15E, neutralizing antibodies were also induced by immunization with the surface envelope protein gp70. PERVs represent a special risk for xenotransplantation using pig tissues or organs since they are integrated in the genome of all pigs and infect human cells and a vaccine may protect from transmission to the recipient. To investigate the effect of simultaneous immunization with both proteins in detail, a study was performed in hamsters. Gp70 and p15E of PERV were produced in *E. coli*, purified and used for immunization. All animals developed binding antibodies against the antigens used for immunization. Sera from animals immunized with p15E recognized epitopes in the MPER and the fusion peptide proximal region (FPPR) of p15E. One MPER epitope showed a sequence homology to an epitope in the MPER of gp41 of HIV-1 recognized by broadly neutralizing antibodies found in HIV infected individuals. Neutralizing antibodies were detected in all sera. Most importantly, sera from animals immunized with gp70 had a higher neutralizing activity when compared with the sera from animals immunized with p15E and sera from animals immunized with gp70 together with p15E had a higher neutralizing activity compared with sera from animals immunized with each antigen alone. These immunization studies are important for the development of vaccines against other retroviruses including the human immunodeficiency virus HIV-1.

## Background

A vaccine is the best protection from an infection with retroviruses including the human immunodeficiency virus HIV-1. Since retroviruses integrate a DNA copy of their genome into the genome of the target cell, were they may persist undetected from the immune system if not expressed, only vaccines based on neutralizing antibodies can prevent virus infection and integration. Neutralizing antibodies have been found in HIV-1 infected individuals, most of them are directed against the surface envelope protein gp120, and only some against the transmembrane envelope protein [1,2]. Two monoclonal antibodies binding to a highly conserved domain in the membrane proximal external region (MPER) of gp41, designated 2F5 and 4E10, have been shown to neutralize up 95% of HIV, however, all attempts to induce such broadly neutralizing antibodies failed until now (for review see [3]). In contrast to HIV-1 it seems easy to induce MPER-specific neutralizing antibodies against gammaretroviruses such as the porcine endogenous retroviruses (PERVs), the feline leukemia virus (FeLV), and the Koala retrovirus, KoRV [4-9].

PERVs pose a special risk when xenotransplantation using pig cells, tissues or organs will be performed in order to overcome the shortage of human allotransplants [10]. PERVs are present in the genome of all pigs, can be released by normal pig cells and infect human cells in vitro (for review see [11]). Although in all preclinical and clinical xenotransplantations as well as in infection experiments, no transmission of PERV was observed, additional safety strategies are under consideration among them vaccination of the human recipient [11]. We recently reported neutralizing antibodies after immunization with the transmembrane envelope protein p15E of PERV in goats [4] and rats [12]. The sera recognized epitopes in the fusion peptide proximal region (FPPR) and the MPER of p15E. When immunizing with the surface envelope protein gp70 much higher titers of neutralizing antibodies were induced compared with p15E [12]. Since studies in rats were hampered by the high prevalence of preexisting antibodies against p15E, hamsters without such preexisting antibodies against p15E were immunized and the effect of simultaneous immunization with p15E and gp70 was analyzed. Immunizing hamsters with p15E resulted in neutralizing antibodies showing a similar epitope pattern as described when immunizing other species. Immunizing hamsters with gp70 also induced higher levels of neutralizing antibodies when compared with the immunization with p15E and immunizing with both envelope proteins resulted in higher titers of neutralizing antibodies compared with the immunization with one antigen alone.

## Results

### Binding antibodies after immunization with p15E and gp70 of PERV

**Figure 1:**
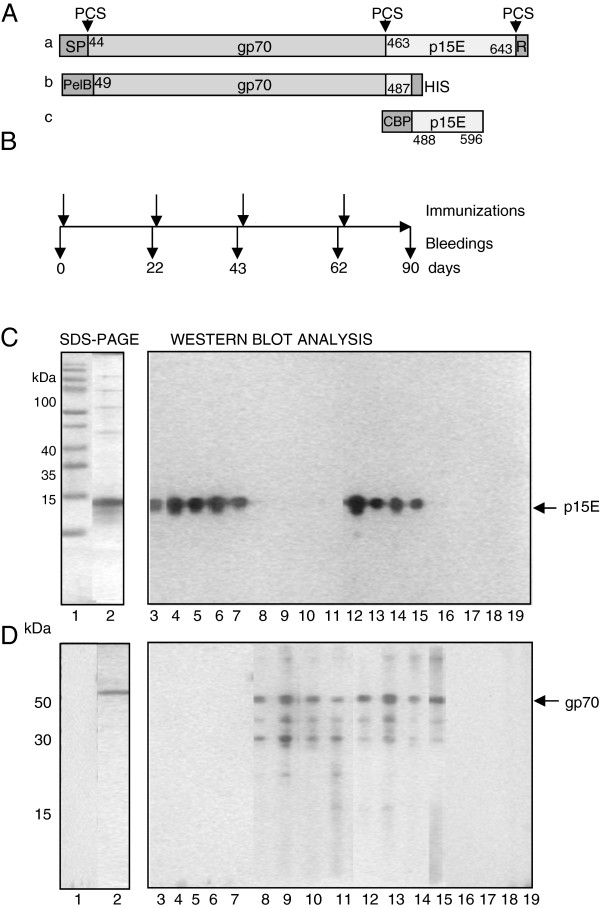
**Antigens, immunization schedule and analysis of the immune sera.** (**A**) Schematic presentation of the viral envelope proteins and antigens used for immunization. (a) Precursor envelope protein of PERV-A (numbering according accession number AJ133817), protease cleavage sites (PCS) are marked with arrow heads, SP - signal peptide. R - R peptide. (b) Recombinant gp70 as expressed using vector pET22b(+) with an N-terminal pelB leader sequence promoting translocation to the periplasma and a C-terminal His-tag. (c) Recombinant p15E with N-terminal fused calmodulin binding protein (CBP) as expressed using the vector pCal-n. (**B**) Immunization schedule indicating the time of immunization and bleedings. (**C**) SDS-PAGE of the purified p15E (12 kDa) and (**D**) gp70 (54 kDa) and Western blot analyses of the immune sera using p15 (**C**) and gp70 (**D**) used for immunization. The fragile gp70 showed smaller molecules due to proteolysis. Lane 1 – marker proteins (not shown in D), lane 2 – purified p15E (**C**) or gp70 (**D**), lane 3–7, sera from animals immunized with p15E, one serum was tested twice, lane 8–9, sera from animals immunized with gp70, lane 12–15, sera from animals immunized with p15E and gp70, lane 16–19, sera from control animals immunized with adjuvant.

**Figure 2:**
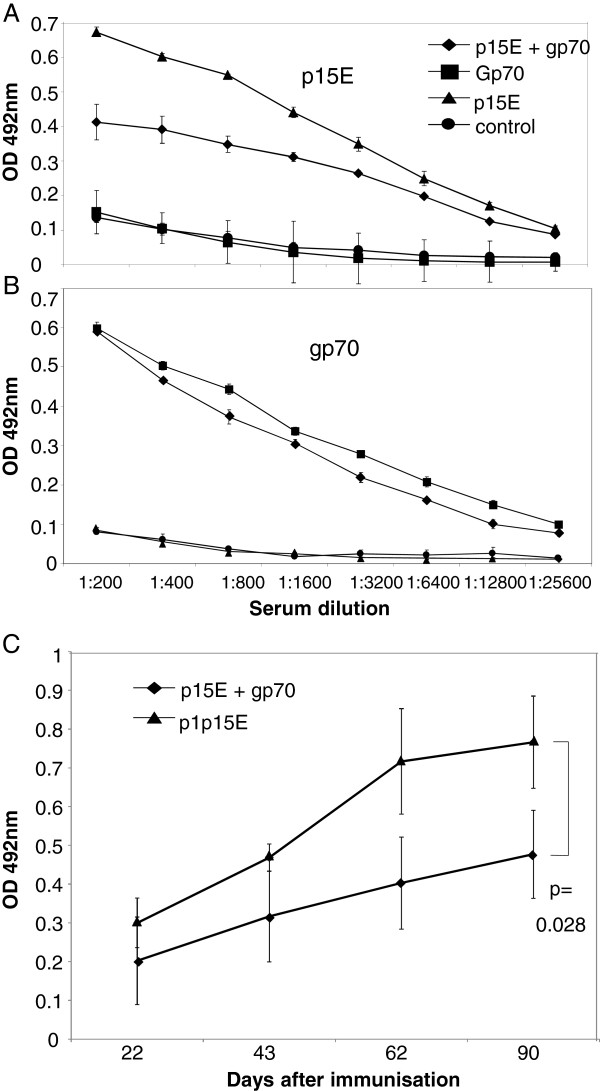
**ELISA of the sera from immunized animals.** (**A, B**) Titration of the sera from animals immunized with p15E, gp70, p15E and gp70 and control animals using recombinant p15E (**A**) and gp70 (**B**) as antigen. The median of all four animals and the corresponding standard deviations are shown. (**C**) Kinetics of induction of antibodies binding to p15E in animals immunized with p15E or p15E and gp70. The dilution of the serum was 1:200. At day 62 and 90 the difference is significant (p=0.028).

In order to induce neutralizing antibodies, the transmembrane envelope protein p15E and the surface envelope protein gp70 of PERV (Figure 1A) were expressed in *E. coli*, purified (Figure 1B) and hamsters were immunized (Figure 1B). Specific binding antibodies were found using Western blot analysis and ELISA in all immune sera (Figure 1C, Figure 2). Although the animals in the group immunized with p15E alone and the animals immunized with p15E and gp70 together received identical amounts of p15E, it remains unknown why the response against p15E is higher in the first group. Investigations of the kinetics showed that already after the first immunization relatively high titers of binding antibodies were observed, which gradually increased after each immunization (Figure 2B).

### Analyzes of the epitopes recognized by antibodies specific for p15E of PERV

**Figure 3:**
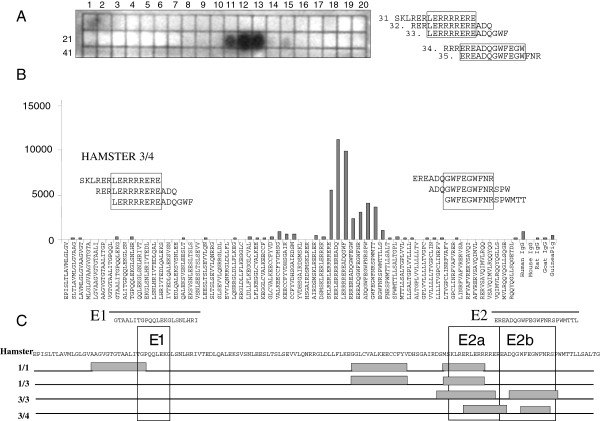
**Epitope mapping of the immune sera using two different methods.** (**A**) Example of an epitope mapping using membranes: serum from hamster 1/3, immunized with p15E. 15-mer peptides overlapping by 12 residues were fixed on the membrane, stained peptides were identified and epitopes determined. (**B**) Example of an epitope mapping using glass slides and the same peptides as in A. Hamster 3/4 was immunized with p15E and gp70 together. (**C**) Summary of the epitopes recognized by two immune sera from animals immunized with p15E (1/1, 1/3) and two sera from animals immunized with p15E and gp70 (3/3, 3/4). The most common epitopes recognized after immunization of goats, rats, mice, and guinea pigs with p15E [4,12] were framed. The peptides E1 and E2 used for screening of the sera were indicated.

In order to map the epitopes in p15E recognized by the immune sera, two different methods were performed. First, overlapping peptides corresponding to the entire p15E were immobilized on a membrane (Figure 3A), and second, a new method was applied using the same overlapping peptides but immobilized on a glass chip (Figure 3B). Both methods identified the same epitopes. In the case of the serum from animal 1/3 immunized with p15E alone, the only epitopes were localized in the MPER of p15E. The localization of the epitopes is similar to the localization described for epitopes induced by immunization with p15E in goats and rats [13]. One of the epitopes in the MPER (GWFEG**WFN**R) is similar in localization and sequence with the epitope of the monoclonal antibody 4E10 (N**WFN**IT, identical amino acids in bold) (Figure 3). 4E10 had been isolated from a HIV-1 infected individual and neutralizes up to 95% of all HIV-1 [14,15]. Despite this limited sequence homology the anti-p15E antibodies and 4E10 did not cross-react and not cross-neutralize (not shown). A screening for binding antibodies using the peptides E1 and E2 (Figure 3C) was not succesful, obviously the peptides were too short to bind the antibodies.

### Neutralizing antibodies after immunization with p15E and gp70 of PERV

**Figure 4:**
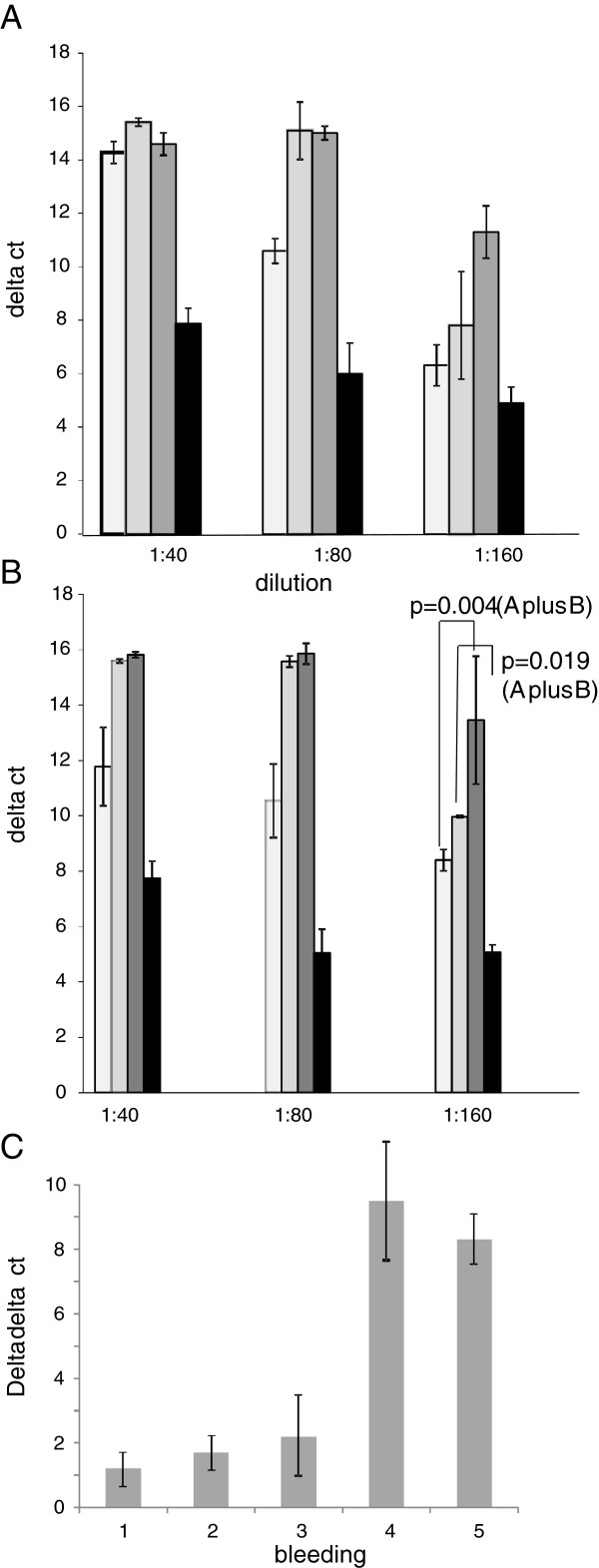
**Neutralizing activity of pooled sera.** (**A**) Sera from animals immunized with p15E (white columns), gp70 (gray columns), p15E and gp70 (dark gray columns) and control animals (black columns) were pooled and analyzed. The delta ct mean of three experiments and the corresponding standard deviation are shown. (**B**) Shown are the results of a second experiment and the Student´s *T*-test evaluation of both experiments. (**C**) Increase in the neutralization titer analyzing the pooled sera from 4 animals immunized with gp70 and p15E. 1 – preimmune serum, 2 –first bleeding 22 days after the first immunization, 3 –bleeding after the second immunization, 4 –bleeding after the third immunization. 4 – bleeding after the fourth immunization.

To analyze the neutralizing activity of the sera a neutralization assay based on the measurement of viral DNA after transcription of the viral genomic RNA by the reverse transcriptase using a real-time PCR was performed and neutralizing activity was found in all immune sera, but not in the preimmune sera and in the sera from control animals immunized with adjuvant alone. The titers of neutralizing activity were higher in sera from animals immunized with gp70 when compared with sera from animals immunized with p15E and the best neutralization was achieved when both envelope protein were used simultaneously (Figure 4A, Figure 4B). Taking both experiments together, the differences were significant (p=0.004 for the difference p15E and gp70 plus p15E; p=0.019 for the difference gp70 and gp70 plus p15E). Investigation of the kinetics showed that the neutralizing activity was still low after two immunizations but increased significantly after the fourth immunization, indicating that multiple immunizations are required to achieve a strong neutralization (Figure 4C). Purified IgG from the immune sera had the same neutralizing effect as the whole serum, indicating that antibodies are responsible for it.

## Discussion

Immunizing for the first time hamsters with the transmembrane envelope protein p15E of PERV resulted in neutralizing antibodies recognizing epitopes in the MPER. These data show that immunization with p15E in all species tested so far resulted in neutralizing antibodies and in antibodies recognizing similar epitopes [4,12]. Neutralizing antibodies and antibodies recognizing the MPER and FPPR had been also induced immunizing with p15E of the feline leukemia virus (FeLV) [5-8], and the koala retrovirus (KoRV) [9]. In the case of the FeLV it was shown that these antibodies protected cats *in vivo* from antigenemia [8]. Whereas in the case of these gammaretroviruses neutralizing antibodies against the transmembrane envelope protein could be easily induced, all attempts to obtain antibodies such as 2F5 and 4E10 broadly neutralizing HIV-1 failed [1-3,16]. In addition, attempts to induce neutralizing antibodies against HIV-2 [17], the feline foamyvirus (FFV) [18], and the primate foamy virus (PFV) (our unpublished data) immunizing with the transmembrane envelope protein also failed. There are some major differences between the transmembrane envelope proteins p15E of the gammaretroviruses and those of the lenti- and foamyviruses. The p15Es are not glycosylated whereas the transmembrane envelope proteins gp41 of HIV-1, gp36 of HIV-2, and gp48 of the foamyviruses are all glycosylated. Whether glycosylation is important for the interaction of the MPER and the FPPR when the N-terminal helical region (NHR) and the C-terminal helical region CHR of the transmembrane envelope proteins of lenti- and foamyviruses interact during infection remains unclear. There is evidence that in the case of HIV-1 MPER and FPPR are in closed proximity at certain moments of the infection process [19-21] and that the presence of a peptide corresponding to the FPPR increases the binding of 2F5 to a peptide containing its epitopes [13].

The neutralization assay used is based on real-time PCR measuring viral DNA in the cells. This assay has several advantages: First, it uses the property of retroviruses to transcribe the viral RNA genome into proviral DNA by the viral reverse transcriptase and measures therefore activity of this enzyme. Second, it measures infection, proviral DNA exists only in the cell. Than higher the ct values then less provirus and then better the neutralizing serum worked. Therefore we suggest that this assay is robust. We used the same assay to measure infection by HIV-1 [13]. This neutralization assay is very sensitive and can be used with low-titer viruses such as PERV. To establish an alternative method, e.g. using an ELISA for viral proteins the virus titer is not high enough to quantify virus infection in 96 well plates. Measuring in parallel GAPDH allows screening of the cell viability.

Hamsters have been chosen for several reason: First to analyze the immune response to p15E in a new species, second to use a larger animal than mice to derive more serum for analysis, and third, to avoid the presence of preexisting antibodies against p15E which were observed for a long time in the preimmune serum of rats used for immunization. Obviously these preexisting antibodies were directed against an endogenous rat gammaretrovirus which is closely related to PERV and we assume that the antibodies were cross-reacting. The endogenous retroviruses of the rat are not well studied [22], but a strong homology with murine and feline leukemia viruses and PERV may be expected. Expression of endogenous retroviruses has been described in numerous species under physiological (e.g., immune responses [23-26]) or pathological conditions (e.g., in tumors of animals [27] and man [28]). Since in hamsters no antibodies cross-reacting with PERV proteins were found, these immunization studies could be performed.

When immunizing with gp70 the neutralizing activity is much higher compared to an immunization with p15E alone and immunization with both envelope proteins induced higher titers of neutralizing antibodies (Figure 4). The same observation was made when immunizing rats with the transmembrane envelope protein of FeLV and gp70 of FeLV [7].

Since there are other strategies under development to prevent transmission of PERVs during xenotransplantation such as inhibition of PERV expression by RNA interference [29,30], it is unlikely that a vaccine against PERV will be required. However, immunization with the transmembrane envelope proteins of gammaretroviruses may help to understand the mechanism of neutralization by MPER-specific antibodies, which is still unclear. The neutralizing antibodies may prevent interaction with the lipids in the membrane or – most likely - conformational changes. The data shows that the MPER is important for the infection of all retroviruses and antibodies against the MPER prevent a crucial step in the infection process. In addition, the data suggests that the use of both envelope proteins may be of advantage despite the fact that the surface envelope protein gp120 of HIV-1 is – in contrast to that of the gammaretroviruses – highly variable. Furthermore, the data shows that two or more immunizations may be required to obtain neutralizing antibodies.

## Conclusions

The induction of PERV-specific neutralizing antibodies in different species including hamster suggests that such antibodies may also be induced in primates including man. Since MPER-specific antibodies were found to neutralize HIV-1 and other retroviruses, these studies may be useful to understand the mechanism how these antibodies neutralize and how to induce such MPER-specific broadly neutralizing antibodies. This data also indicate that the MPER is a highly vulnerable target for the neutralization of retroviruses in general.

## Methods

### Cloning and purification of antigens p15E and gp70

The ectodomain of p15E of PERV-A (amino acids 488–596, accession number HQ688786) and a recombinant protein corresponding to gp70 of PERV-A (amino acids 49–487, accession number HQ688785) (Figure 1A) were cloned into the pET-22b(+) expression vector (Novagen, San Diego, CA), expressed in *E. coli* BL21-CodonPlus(DE3)-RP (Stratagene, Amsterdam), and purified by metal chelating affinity chromatography using Ni-NTA (Qiagen) as described [12]. The cloned sequence of gp70 resembles the sequence of gp70 of FeLV, used as the commercial “Leucogen” for vaccination of cats containing a small part of p15E [31]. The p15E (LITGPQQLEKGLSNLHRIVTEDLQALEKSVSNLEESLTSLSEVVLQNRRGLDLLFLKEGGLCVALKEECCFYVDHSGAIRDSMSKLRERLEKRHKEKEAGQGWFEGWFN) is a fusion protein with the calmodulin binding protein (CBP) (MKRRWKKNFIAVSAANRFKKISSSGALLVPR). It theoretical molecular mass is 16.3 kDa, however it is always detected at 12 kDa (Figure 1C). The molecular mass of the recombinant gp70 is 54 kDa (Figure 1D).

### Immunization

Hamsters (Charles River) were immunized with 300 μg of p15E, gp70 or both. In the last case, gp70 and p15E were immunized in different parts of the body. The proteins were emulsified in complete Freund’s adjuvant intramuscularly and subcutaneously (i.m. 50μl, s.c. 700μl). The control animals were immunized with adjuvant and PBS. The immune response was boosted by second and third immunizations using incomplete Freund’s adjuvant (Figure 1B). IgGs were concentrated using Vivapure Q Mini spin columns (Vivascience). Control animals were immunized with adjuvant only.

### Peptides, Western blot, and ELISA

Peptides E1(484–505) GTAALITGPQQLEKGLSNLHRI and E2(583–604) EREADQGWFEGWFNRSPWMTTL (Figure 3C) were synthesized by Gene Cust, Dulange, Luxembourg. Western blot and ELISA were performed as described before [12] using the recombinant proteins gp70 and p15E. 0.2 μg/well recombinant proteins were used for ELISA; sera were diluted 1:200 to 1:25600. A secondary antibody labeled with HRP was used for ECL detection. Each serum was titrated and the mean of each group is shown in Figure 1A, B, C).

### Epitope mapping

The entire p15E of PERV (130 amino acids) was synthesized as a cellulose-adsorbed peptide spot library of 15-mer peptides overlapping by 12 amino acids or a glass based chip with the same peptides (JPT Peptide Technologies, Germany) using standard protocols of the supplier. Sera were diluted 1:1000 and binding was detected using a chemiluminescence detection solution (ECL, Amersham Pharmacia Biotech) or bound antibodies were detected using a DyLight 649 conjugated AffiniPure goat anti-rat IgG antibody and read at a wavelength of 635 nm in a GenePix 4000 microarray scanner (Molecular Devices, USA). The data were analyzed using the GenePix Pro software. Epitopes were defined as central amino acids shared by all peptides recognized by the antiserum (see Figure 3A, B).

### Neutralization assay

The neutralization assays were performed as described using virus-containing cell-free supernatants produced by human embryonic kidney 293 cells infected with PERV/5° [12]. This virus is a recombinant human-tropic PERV-A/C repeatedly passaged on human cells which was associated with increased titers and genetic alterations in its long terminal repeats (LTR) [32]. 100 μl uninfected 293 cell (1.5 × 10^5^/ml) were seeded in 96 well plates and incubated for 4 h at 37°C in 5% (v/v) CO_2_. Sera were decomplemented by heat inactivation (30 min at 56°C) and 20 μl were mixed with 80 μl of a PERV dilution, incubated for 30 min at 37°C and added to the 293 cells. Virus dilutions resulting in stable Ct values (25 to 27) were considered as optimal for the neutralization assay. After incubation for 72 h at 37°C cells were examined by light microscopy for viability and the medium was removed. Cells were lysed by heating at 95°C for 30 min, freezing at −20°C for 6 h, and incubation with lysis buffer (nuclease free water containing 0.2 mg/ml proteinase K and 10% (v/v) 10×PCR-buffer) at 60°C for at least 3 h. Proteinase K was heat inactivated (30 min at 95°C). For quantification of PERV proviral DNA the primers gag-for and gag-rev located in the *gag* gene and a specific PERV-gag probe (Table 1) were used in a duplex real-time PCR. The reference gene GAPDH was amplified with the primers GAPDH-for and GAPDH-rev and quantified using a GAPDH-probe (Table 1). The 22 μl reaction mixture consisted of 1x PCR buffer with 1.5 mM MgCl_2_, 0.5 μM each of dATP, dCTP, dGTP, dTTP, 5 pmol of each primer, 5 pmol of probe, 1.25 U AmpliTaq Gold^®^ polymerase and 3 μl lysate. The thermal cycling conditions used were 10 min at 95°C followed by 50 cycles of 1 min at 95°C, 1 min at 59°C and 30 s at 72°C in a Stratagene MX4000 machine. Neutralization was defined as reduction of provirus integration in the presence of immune serum and calculated as (ct value of PERV - ct value of GAPDH) in the presence of serum - (ct value of PERV - ct value of GAPDH) in the absence of serum. The ct values of GAPDH were identical in all samples, indicating absence of toxic effects of the sera. In addition the delta delta ct values were determined as described [33].

**Table 1:** Primers and probes

**Primer/probe**	**Sequence 5’-3’**	**Location**	**Accession Nr.**
hGAPDH-for	GGCGATGCTGGCGCTGAGTAC	365.385	AF261085
hGAPDH-rev	TGGTCCACACCCATGACGA	495.513	AF261085
hGAPDH-probe	HEX-TTCACCACCATGGAGAAGGCTGGG-BHQI	407.430	AF261085
PERV-gag-for	TCCAGGGCTCATAATTTGTC	1213.1232	AJ293656
PERV-gag-rev	TGATGGCCATCCAACATCGA	1289.1308	AJ293656
PERV-gag-probe	FAM-AGAAGGGACCTTGGCAGACTTTCT-BHQ1	1244.1267	AJ293656

## Competing interests

The authors declare that they have no competing interests.

## Authors’ contributions

DM and DK carried out the production of the antigens and neutralization assays, C-MS performed the Western blot assays, ELISA and epitope mapping, JD organized and supervised the study and wrote the manuscript, all authors read and approved the manuscript.

## References

[B1] Walker LM Huber M Doores KJ Falkowska E Pejchal R Julien JP Wang SK Ramos A Chan-Hui PY Moyle M Mitcham JL Hammond PW Olsen OA Phung P Fling S Wong CH Phogat S Wrin T Simek MD Koff WC Wilson IA Burton DR Poignard P Protocol G Principal Investigators Broad neutralization coverage of HIV by multiple highly potent antibodies Nature 2011 477 466 470 10.1038/nature10373 21849977 PMC3393110

[B2] Wu X Yang ZY Li Y Hogerkorp CM Schief WR Seaman MS Zhou T Schmidt SD Wu L Xu L Longo NS McKee K O'Dell S Louder MK Wycuff DL Feng Y Nason M Doria-Rose N Connors M Kwong PD Roederer M Wyatt RT Nabel GJ Mascola JR Rational design of envelope identifies broadly neutralizing human monoclonal antibodies to HIV-1 Science 2010 329 856 861 10.1126/science.1187659 20616233 PMC2965066

[B3] Montero M van Houten NE Wang X Scott JK The membrane-proximal external region of the human immunodeficiency virus type 1 envelope: dominant site of antibody neutralization and target for vaccine design Microbiol Mol Biol Rev 2008 72 54 84 10.1128/MMBR.00020-07 18322034 PMC2268283

[B4] Fiebig U Stephan O Kurth R Denner J Neutralizing antibodies against conserved domains of p15E of porcine endogenous retroviruses: basis for a vaccine for xenotransplantation? Virology 2003 307 406 413 10.1016/S0042-6822(02)00140-X 12667808

[B5] Langhammer S Fiebig U Kurth R Denner J Neutralising antibodies against the transmembrane protein of feline leukaemia virus (FeLV) Vaccine 2005 23 3341 3348 10.1016/j.vaccine.2005.01.091 15837241

[B6] Langhammer S Hubner J Kurth R Denner J Antibodies neutralizing feline leukaemia virus (FeLV) in cats immunized with the transmembrane envelope protein p15E Immunology 2006 117 229 237 10.1111/j.1365-2567.2005.02291.x 16423059 PMC1782217

[B7] Langhammer S Fiebig U Kurth R Denner J Increased neutralizing antibody response after simultaneous immunization with Leucogen and the feline leukemia virus transmembrane protein Intervirology 2010 54 78 86 20829603 10.1159/000318892

[B8] Langhammer S Hübner J Jarrett O Kurth R Denner J Immunization with the transmembrane protein of a retrovirus, feline leukemia virus: absence of antigenemia following challenge Antiviral Res 2011 89 119 123 10.1016/j.antiviral.2010.11.011 21108970

[B9] Fiebig U Hartmann MG Bannert N Kurth R Denner J Transspecies transmission of the endogenous koala retrovirus J Virol 2006 80 5651 5654 10.1128/JVI.02597-05 16699047 PMC1472152

[B10] Wilson CA Porcine endogenous retroviruses and xenotransplantation Cell Mol Life Sci 2008 65 3399 3412 10.1007/s00018-008-8498-z 18818871 PMC11131834

[B11] Denner J Tönjes RR Infection barriers to succesful xenbotransplantation focusing on porcine endogenous retroviruses Clin Microbiol Rev 2012 25 318 343 10.1128/CMR.05011-11 22491774 PMC3346299

[B12] Kaulitz D Fiebig U Eschricht M Wurzbacher C Kurth R Denner J Generation of neutralising antibodies against porcine endogenous retroviruses (PERVs) Virology 2011 411 78 86 10.1016/j.virol.2010.12.032 21237477

[B13] Fiebig U Eschricht M Schmolke M Kurth R Denner J Mode of interaction between the HIV-1 neutralizing monoclonal antibody 2F5 and its epitope AIDS 2009 23 887 895 10.1097/QAD.0b013e3283292153 19414989

[B14] Zwick MB Labrijn AF Wang M Spenlehauer C Saphire EO Binley JM Moore JP Stiegler G Katinger H Burton DR Parren PW Broadly neutralizing antibodies targeted to the membrane-proximal external region of human immunodeficiency virus type 1 glycoprotein gp41 J Virol 2001 75 10892 10905 10.1128/JVI.75.22.10892-10905.2001 11602729 PMC114669

[B15] Muster T Steindl F Purtscher M Trkola A Klima A Himmler G Rüker F Katinger H A conserved neutralizing epitope on gp41 of human immunodeficiency virus type 1 J Virol 1993 67 6642 6647 7692082 10.1128/jvi.67.11.6642-6647.1993 PMC238102

[B16] Denner J Towards an AIDS vaccine: the transmembrane envelope protein as target for broadly neutralizing antibodies Hum Vaccin 2011 7 4 9 10.4161/hv.7.0.14555 21266839

[B17] Behrendt R Fiebig U Kurth R Denner J Induction of antibodies binding to the membrane proximal external region (MPER) of gp36 of HIV-2 Intervirology 2012 55 252 256 10.1159/000324483 21454955

[B18] Mühle M Bleiholder A Kolb S Hübner J Löchelt M Denner J Immunological properties of the transmembrane envelope protein of the feline foamy virus and its use for serological screening Virology 2011 412 333 340 10.1016/j.virol.2011.01.023 21316070

[B19] Bellamy-McIntyre AK Lay CS Baär S Maerz AL Talbo GH Drummer HE Functional links between the fusion peptide-proximal polar segment and membrane-proximal region of human immunodeficiency virus gp41 in distinct phases of membrane fusion J Biol Chem 2007 282 23104 23116 10.1074/jbc.M703485200 17526486

[B20] Noah E Biron Z Naider F Arshava B Anglister J The membrane proximal external region of the HIV-1 envelope glycoprotein gp41 contributes to the stabilization of the six-helix bundle formed with a matching N' peptide Biochemistry 2008 47 6782 6792 10.1021/bi7023139 18540633 PMC2459237

[B21] Buzon V Natrajan G Schibli D Campelo F Kozlov MM Weissenhorn W Crystal structure of HIV-1 gp41 including both fusion peptide and membrane proximal external regions PLoS Pathog 2010 6 e1000880 10.1371/journal.ppat.1000880 20463810 PMC2865522

[B22] Lee SY Howard TM Rasheed S Genetic analysis of the rat leukemia virus: influence of viral sequences in transduction of the c-ras proto-oncogene and expression of its transforming activity J Virol 1998 72 9906 9917 9811727 10.1128/jvi.72.12.9906-9917.1998 PMC110503

[B23] De Lamarter JF Monckton RP Moroni C Transcriptional control of endogenous virus genes in murine lymphocytes J Gen Virol 1981 52 371 375 10.1099/0022-1317-52-2-371 7288399

[B24] Stoye JP Moroni C Endogenous retrovirus expression in stimulated murine lymphocytes. Identification of a new locus controlling mitogen induction of a defective virus J Exp Med 1983 157 1660 1674 10.1084/jem.157.5.1660 6189943 PMC2187009

[B25] Hirsch MS Phillips SM Solnik C Black PH Schwartz RS Carpenter CB Activation of leukemia viruses by graft-versus-host and mixed lymphocyte reactions in vitro Proc Natl Acad Sci USA 1972 69 1069 1072 10.1073/pnas.69.5.1069 4402535 PMC426630

[B26] Denner J Dorfman N Small virus-like particles in leukosis-like syndrome induced by certain antigens and immunostimulators Acta Biol Med German 1977 36 1451 1458 616161

[B27] Nelson M Nelson DS Spradbrow PB Kuchroo VK Jennings PA Cianciolo GJ Snyderman R Successful tumour immunotherapy: possible role of antibodies to anti-inflammatory factors produced by neoplasms Clin Exp Immunol 1985 61 109 117 4042416 PMC1577247

[B28] Büscher K Hahn S Hofmann M Trefzer U Ozel M Sterry W Löwer J Löwer R Kurth R Denner J Expression of the human endogenous retrovirus-K transmembrane envelope, Rec and Np9 proteins in melanomas and melanoma cell lines Melanoma Res 2006 16 223 234 10.1097/01.cmr.0000215031.07941.ca 16718269

[B29] Ramsoondar J Vaught T Ball S Production of transgenic pigs that express porcine endogenous retrovirus small interfering RNAs Xenotransplantation 2009 16 164 180 10.1111/j.1399-3089.2009.00525.x 19566656

[B30] Semaan M Kaulitz D Petersen B Niemann H Denner J Long-term effects of PERV-specific RNA interference in transgenic pigs Xenotransplantation 2012 19 112 121 10.1111/j.1399-3089.2012.00683.x 22497513

[B31] Marciani DJ Kensil CR Beltz GA Hung CH Cronier J Aubert A Genetically-engineered subunit vaccine against feline leukaemia virus: protective immune response in cats Vaccine 1991 9 89 96 10.1016/0264-410X(91)90262-5 1647576

[B32] Denner J Specke V Thiesen U Karlas A Kurth R Genetic alterations of the long terminal repeat of an ecotropic porcine endogenous retrovirus during passage in human cells Virology 2003 314 125 133 10.1016/S0042-6822(03)00428-8 14517066

[B33] Livak KJ Schmittgen TD Analysis of relative gene expression data using real-time quantitative PCR and the 2(−Delta Delta C(T)) Method Methods 2001 25 402 408 10.1006/meth.2001.1262 11846609

